# Dexamethasone Suppresses Already Low Estrogen Receptor Levels in Meningiomas

**DOI:** 10.3390/ijms27062779

**Published:** 2026-03-19

**Authors:** Judith C. Hugh, Lacey S. J. Haddon, John Maringa Githaka

**Affiliations:** 1 Department of Laboratory Medicine and Pathology, University of Alberta, 116 St & 85 Ave, Edmonton, AB T6G 2R3, Canada; 2 Department of Chemistry, University of Alberta, 116 St & 85 Ave, Edmonton, AB T6G 2R3, Canada; lhaddon@ualberta.ca; 3 Department of Medical Oncology, WVU Cancer Institute, 1 Medical Center Drive, Morgantown, WV 26506, USA; jmg00102@hsc.wvu.edu

**Keywords:** estrogen receptor, meningioma, brain derived estrogen, dexamethasone

## Abstract

Intracranial meningiomas (ICMs) are the most common primary adult brain tumor. They are more frequent in women, respond to female hormones, are associated with breast cancer and are often progesterone receptor-positive (PR+), consistent with hormonal sensitivity. Yet <20% are weakly estrogen receptor-positive (ER+). This work reviews the literature to investigate this paucity of ER by first testing if Dexamethasone (Dex), which has been used since 1984 to reduce peritumoral brain edema, is suppressing ER. Ligand-binding assays after 1984 have shown a significant decrease in any and supra-threshold (>10 fmol/mg) ER+ from 68.5% and 39.6% to 25.5% and 12%, respectively (both *p* < 0.0001). This was confirmed as Dex-related in 93 patients with known Dex exposure (*p* = 0.0075). Immunohistochemical tests after 1984 have shown that 16% (95%CI 8.4–24.4) of ICMs have rare ER+ cells unrelated to PR and pS2 expression, consistent with Dex inhibition of ER transcription activity. Dex suppression of ER may be compounded by lower endogenous ER concentrations in ICMs compared to breast cancer. The difference in intra-tumoral estrogen concentration is proposed as a potential cause for lower ER in ICM. Replacement of Dex and more sensitive ER assays are needed to determine the role of hormones in the causation and treatment of ER+ ICM.

## 1. Introduction

Intracranial meningiomas (ICMs) account for 42.3% of all and 57.4% of non-malignant primary brain and other central nervous system (CNS) tumors, with 52,690 cases projected for 2026 in the USA [1]. Their incidence increases with age, with incidental tumors detected in 1.5–3% of the population over 60 years old [2,3]. Although 81% of these tumors are considered benign (CNS WHO grade 1) with a 10-year survival rate of 84% [1], over 20% may recur even if completely excised [4]. The age-adjusted rate per 100,000 and average annual incidence of CNS WHO grade 1 ICM is higher in Black (3.39; 1458) compared to white (2.85; 7603) populations, with increasing disparity with higher tumor grade [1]. Environmental risks include ionizing radiation, increased body mass index, and exogenous synthetic progestins, particularly cyproterone acetate [5]. Although several mutations have been linked to ICM, the major genetic risk factor is Neurofibromatosis 2-related schwannomatosis (NF2-SWN) [5]. NF-2 mutations have also been reported in sporadic tumors of all grades but are increased in higher grades [6].

**Figure 1:**
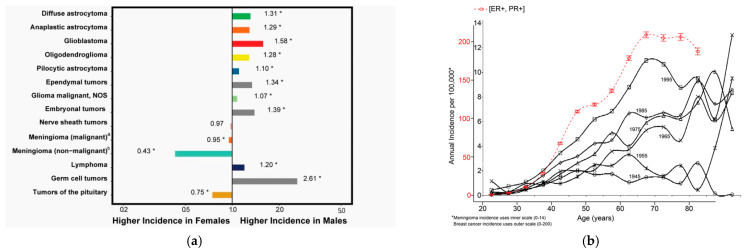
(**a**) Incidence rate ratios by sex (males:females) for selected primary brain and other central nervous system tumor histopathologies ^a, b^. * Incidence rate is significantly different between groups at the *p* < 0.05 level. ^a^ Malignant meningioma roughly correlates to CNS WHO grade 2 and 3. ^b^ Non-malignant meningioma roughly correlates to CNS WHO grade 1. Originally published by Price M. et al. 2025, Figure 21 [1]. Used with permission from Oxford University Press, License no. 6147690316850. (**b**) Comparison of age-specific incidence of ER+PR+ breast cancers and meningiomas. Superimposition of ER+PR+ breast cancers in white women (red dotted line) from Surveillance Epidemiology and End Results (SEER17 data from 2004 to 2008) using the outer *Y*-axis and meningiomas in Denmark 1993–1997 (solid line with open squares) using the inner *Y*-axis scale. (Originally published by Christensen H. et al. 2003, Figure 4 [8], modified by the addition of the SEER curve. Used with permission from Wolters Kluwer Health Inc. License no. 6151570270997). (ER+PR+ curve extracted from Gleason, M. et al. 2012, Figure 3a [9] under Creative Commons Attribution License of unrestricted use.).

Meningiomas show a singular female predominance, with a male-to-female ratio for WHO grade 1 tumors of 0.43 [1] (Figure 1a) and a 10-fold increased risk of subsequent breast cancer in female patients with meningioma [7]. The age-specific incidence of meningiomas in 1993–1997 [8] shows a striking resemblance to breast cancers that are estrogen receptor-positive (ER+) and progesterone receptor-positive (PR+) [9] as both tumors have an undulation known as Clemmesen’s hook [10] during major hormonal fluxes in the peri-menopausal period (Figure 1b). Similarly, there can be transient worsening of symptoms during late pregnancy [11] and in the luteal phase of the menstrual cycle [12], periods characterized by increased estrogen and progesterone. These findings, combined with the presence of PR in the majority of tumors [13,14], a known transcription product of ER [15], are highly suggestive of a hormonal etiology for many ICMs.

However, the importance of hormones in the pathogenesis and treatment of ICM has largely been discounted because of the paucity of ER in these tumors [13,14] and the ineffectiveness of initial trials of anti-estrogen agents [16,17]. This paper investigates potential causes for the low ER level in ICM by first testing the hypothesis that the routine use of Dexamethasone (Dex) for peritumoral brain edema (PTBE) is suppressing ER levels and ER signaling and secondly by proposing a hypothesis whereby ICMs have lower endogenous levels of ER than breast cancer. The aim of this paper is to increase the understanding of how hormones affect meningiomas so that ultimately hormonal therapy can be part of a precision medicine approach to treatment for selected patients.

## 2. Dexamethasone and ER

Dex is a synthetic corticosteroid that has low mineralocorticoid activity but glucocorticoid activity that is 30X more potent than endogenous cortisol. It binds exclusively to albumin and is metabolized through the hepatic cytochrome p450 system with subsequent renal excretion of the inactive metabolites. Dex reaches peak concentration within 1–1.5 h and has a plasma half-life of 2–4 h but an extended biologic half-life of 34–54 h [18]. When given immediately before surgery, the peak levels correspond to the estimated time of tumor resection [18]. After a 1961 landmark paper [19] showing significant improvement in neurological symptoms in 13/14 brain tumor patients with PTBE, it became a standard treatment for all neurosurgical tumor resections, and by 1984 most ICMs in the USA were treated with perioperative Dex [20]. A major mechanism by which Dex reverses PTBE is through the inhibition of vascular endothelial growth factor (VEGF), a potent vasodilator and angiogenic stimulus [21]. However, beginning in 2015 [22], Dex administration during radiotherapy was found to correlate with a worse outcome in glioblastoma patients [23,24], most likely due to the inhibition of cell cycle genes, creating a more radioresistant state [25]. Other potential adverse effects of Dex include immunosuppression due to lymphocyte apoptosis, decreases in both innate and adaptive immunity (reviewed in [26]) and an adverse effect on immune checkpoint therapies that target PD-L1 [27]. This latter effect might be significant for ICM as PD-L1 expression increases in ICM progression to higher grades [28]. A clinical trial is currently underway to restrict the use of Dex in newly referred glioblastoma patients (NCT05266977).

38% to 67% of ICM patients [29] have PTBE which is primarily caused by tumor cell VEGF secretion into peritumoral tissue [30]. However, because meningiomas arise from the extracerebral arachnoid membrane, VEGF only induces PTBE if the tumor has breached the cerebral cortex and elicited a secondary cerebral–pial blood supply [31]. This is thought to underly the reduced efficacy of Dex in PTBE in ICM [32,33,34].

Of particular importance to ICM, Dex also downregulates ER. Multiple in vitro studies in diverse cell types [35,36,37,38,39,40] have found that within 1 h of Dex administration, there is a reduction in nuclear estrogen–ER binding coupled with a decrease in ERα levels by 24 h. Karmakar et al. [41] and later Yang et al. [42] using the MCF-7 ER+ breast cancer cell line found that Dex bound to glucocorticoid receptors (GRs) was recruited to DNA sites that were already occupied by ER bound to estrogen. The DNA-binding domain of GR initiated a protein–protein interaction with ERα, impairing ER binding, destabilizing the ERα–transcription factor complex, leading to the inhibition of transcription and eviction of the liganded ER homodimers and the inhibition of estrogen-induced signaling [41,42].

Since GRs are present in the majority of meningiomas [43,44] and Dex-GR complexes can decrease ER transcriptional signaling [41,42] and lower levels of ER in vitro [35,36,37,38,39,40], the hypothesis tested in this work is that perioperative Dex given to reduce PTBE is reducing ER levels and ER signaling in ICM.

## 3. Review of Estrogen Receptor Testing in Intracranial Meningiomas

To test whether Dex can downregulate ER and ER signaling in ICM, the ER assay literature is reviewed below. Assays for ER in ICM used the techniques and cut-off points established in breast cancer. The earliest tests were ligand-binding assays (LBAs), which detected the binding of radiolabeled 17 β-estradiol or estradiol analogues in fresh frozen tissue, beginning in 1979 with Donnell et al. [45] who reported estradiol binding in five of six ICM patients. Using a recent systemic review [14] as the initial basis for article retrieval, the results of ER assays will be compared before and after 1984 to investigate the effect of routine perioperative Dex on ER detection and concentration. Subsequently, in the late 1980s, as commercial antibodies against ER became available, immunohistochemical tests (IHC) that specifically detect ER in tumor cells became the standard assay for hormone receptor testing in breast cancer and ICM. Using a second systemic review [13] as the basis for article retrieval, the IHC literature is reviewed for evidence of Dex alteration of ER signaling.

### 3.1. Ligand-Binding Assays

In 2023, Miyagishima et al. [14] published a large systematic review and meta-analysis of gonadal steroid hormone receptors in meningioma. Following Preferred Reporting Items for Systematic Reviews and Meta-analyses (PRISMA)-IPD 2015 guidelines and using English-language publications indexed in MEDLINE PubMed for ER, PR and androgen receptors, they analyzed data from 6092 tumors in 114 investigations published between 1 January 1951 and 31 December 2020. They investigated the proportion of positive tumors as well as associations between hormone receptor and tumor characteristics. In their review, the IHC threshold for hormone receptor-positive samples was defined as “any staining”, whereas for LBA, only tumors with ligand binding ≥10 Femtomoles per milligram of cytoplasmic protein (fmol/mg) were considered positive. They found that the proportion of ICMs containing receptors was 0.76 for PR and 0.06 for ER. PR status was associated with meningothelial histology, skull base location, increased age, and WHO Grade 1, suggesting that hormones may be important in a defined subset of low-grade meningiomas. However, no similar relationships were found for ER. They noted that ER detection was higher with LBAs, at 0.11 (95% CI 0.06–0.20), than with IHC tests, at 0.03 (95% CI 0.01–0.09). Within LBAs, the year of publication had a significant association with ER expression (1.01, *p* < 0.00001).

**Figure A1:**
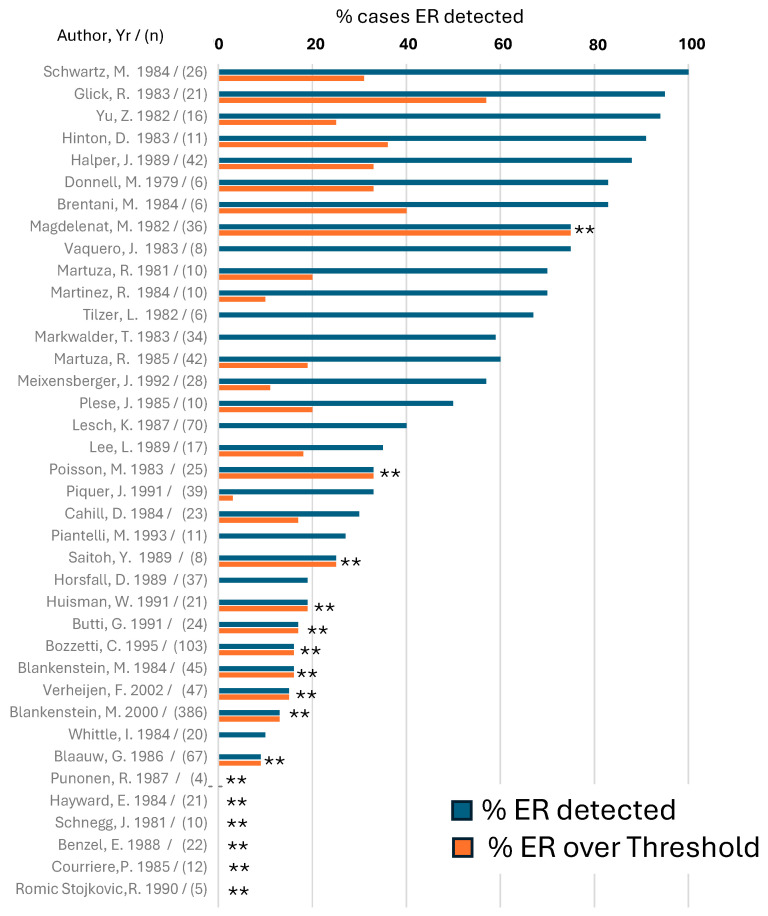
Data were extracted from the tables in each reference [20,45,46,47,48,49,50,51,52,53,54,55,56,57,58,59,60,61,62,63,64,65,66,67,68,69,70,71,72,73,74,75,76,77,78,79,80,81]. Any non-zero assay result was counted as ER detected. ER over threshold represents cases whose assay value exceeded a listed threshold. ** Data only available for cases that exceeded threshold.

To test whether the effect of the publication year for LBA was due to the routine use of perioperative Dex, the ER assay data reviewed by Miyagishima et al. [14] were re-extracted and analyzed by year of publication using studies from 1984 onwards as patients who were most likely Dex-exposed [20]. For this analysis, the studies detailed in Miyagishima’s Supplemental Figure S8 [20,46,47,48,49,50,51,52,53,54,55,56,57,58,59,60,61,62,63,64,65,66,67,68,69,70,71,72,73,74] were supplemented with eight other published LBA studies, with most reporting ER assay values as fmol/gm of tumor [45,75,76,77,78,79,80,81], resulting in a total of 38 studies (1341 patients) with a mean percentage ER positivity of 15.86 (95% CI 10.24–21.47). Data were also extracted for any case which reported a non-zero value for ER indicating the presence of detectable ER (*Any ER*) (see Figure A1). The data retrieval process for *Any ER* is similar to the approach used by Miyagishima et al. [14], using the IHC assay, where any staining was considered positive. These *Any ER* cases are distinguished from the supra-threshold or “ER+” cases with ER assay values over the thresholds of ≥10 fmol/mg of cytoplasmic protein or 100 ≥ fmol/gm of tumor used in breast cancer.

**Figure 2:**
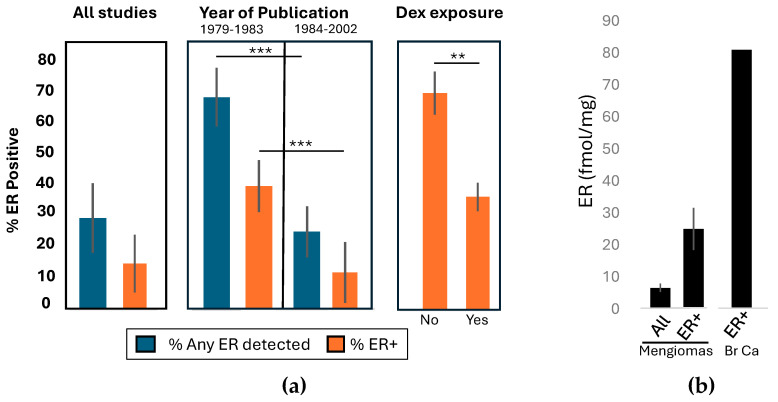
(**a**) The mean and standard deviation for percentage of *Any ER* detected and ER+ cases. The first panel illustrates the results from the entire series while the second panel divides these by year of publication. The third panel compares the %ER+ for 93 patients with known Dex exposure (see Table A1). ** *p* < 0.01. *** *p* < 0.0001. (**b**) The concentration of ER (fmol/mg) in meningiomas and breast cancer. Assay values are taken from 24 studies [20,46,48,50,51,52,53,54,55,56,57,58,59,60,61,62,64,65,66,67,69,75,77,79] that used fmol/mg. The following three outlying values were excluded (1270 fmol/mg from [58], 498 and 208 fmol/mg from [46]).

In this series, the percentage of ICMs with ER+ values was 15% ± 9.4 (1 SD) (202/1341), with 30% ± 11.4 (1 SD) having *Any ER* (Figure 2a, first panel). When the data were subdivided by year of publication (Figure 2a, second panel), the percentage of ER+ ICM in the earlier (1979–1983) compared to the later (1984–2002) cohort was 39.6% (59/149) vs. 12% (143/1192), respectively, (Yates’ continuity-corrected chi-square (χ^2^) test, *p* < 0.0001). Similarly, the percentage of tumors containing *Any ER* was higher in studies prior to 1984 compared to studies after 1984, at 68.5% (102/149) vs. 25.5% (304/1192) (Yates’ continuity-corrected chi-square (χ^2^) test, *p* < 0.0001). This confirms that the year of publication was a significant factor in ER positivity. Importantly, four studies [46,47,76,78] published around 1982–1984 recorded assay values for ER+ cases and specified whether Dex had been used in a total of 93 patients (see Table A1). These show a significant decrease in %ER+ from 69.2% to 35.8% (Yates’ continuity-corrected chi-square (χ^2^) *p*-value of 0.0075), consistent with a 50% decrease in ER positivity after Dex exposure (Figure 2a, third panel).

Finally, the concentration of ER was calculated for 90 ER+ patients and 419 patients from 24 papers [20,46,48,50,51,52,53,54,55,56,57,58,59,60,61,62,64,65,66,67,69,75,77,79] that used the dextran-coated charcoal assay and expressed their results in fmol/mg of cytosolic protein; most of these patients were likely Dex-exposed. For ER+ tumors, the mean ± standard error of the mean (SEM) was 25.1 ± 6.7 fmol/mg. Considering all 419 patients, the ER concentration was 6.3 ± 1.3 fmol/mg. The latter value is similar to the mean value of 4 fmol/mg from 386 tumors tested by a single lab [68]. Assuming that this level represents 50% of the ER level without Dex, the original ER concentration might be approximately 8–12 fmol/mg. For comparison purposes, a contemporary study of 52 ER+ metastatic breast cancer patients [82] had a mean ER level of 80.6 fmol/mg (Figure 2b). This suggests that without Dex exposure, ICMs have an endogenous ER concentration that is ~15% of the average breast cancer concentration.

In summary, this analysis finds that LBAs detected a low level of ER in 60–70% of meningiomas but this decreased by at least 50% after the administration of perioperative Dex became routine around 1984.

### 3.2. Immunohistochemistry Assays for Estrogen Receptor

**Figure 3:**
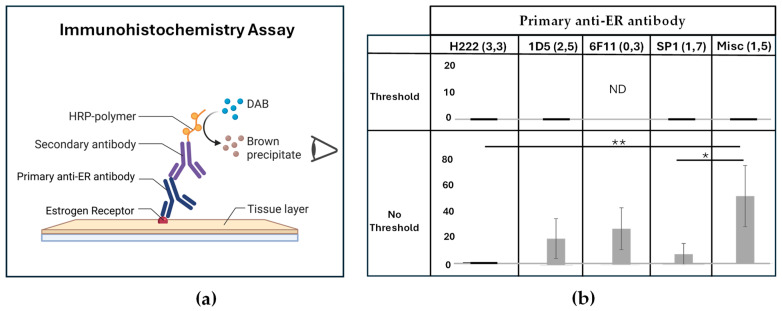
(**a**) The mechanism of the immunohistochemistry assay. HRP—horseradish peroxidase, DAB—3,3′-diaminobenzidine. Created in BioRender. Hugh, J. (2026). https://BioRender.com/ca4epm7, accessed on 20 February 2026. (**b**) The percentage of cases positive for ER in IHC assays by primary anti-ER antibody and the use of a threshold. “Misc” refers to miscellaneous or non-standard antibodies. The number of studies with each antibody is indicated in brackets, with a comma separating the studies by their use of a quantitative threshold (Threshold, No Threshold). Pairwise comparisons between H222, 1D5, 6F11 and SP1 antibodies are not significant. Significant differences between “No Threshold” groups by non-parametric Kruskal–Wallis (*p* = 0.0025) are due to differences shown between H222 and Misc (** *p* = 0.004) and SP1 and Misc (* *p* = 0.026), according to Dunn’s post hoc analysis.

ER determinations in ICM followed breast cancer in shifting from LBAs to IHC tests in the late 1980s (see Figure 3a), meaning that virtually all IHC assays involved Dex-exposed patients. The ability to use archived paraffin block ICM material in IHC assays greatly increased the average number of cases per study (67 cases/study) compared to LBA (25 cases/study). This review of IHC ER results allows for confirmation of the lower level of ER after Dex, while the larger number of cases per study allows for an assessment of ER signaling function.

**Figure A2:**
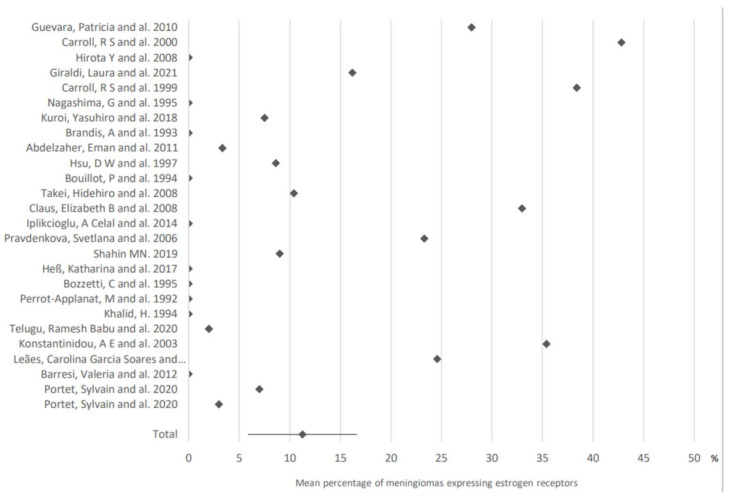
The expression of estrogen receptors in meningiomas. The results are expressed as the mean and 95% confidence interval. The number of articles analyzed related to the topic was 26. The number of included cases (total for all studies; minimum; maximum in each study) was 1528 (21;161). From Figure 2 of Agopiantz et al. [13]. Used through Creative Commons Attribution (CC BY) license.

While both Miyagishima et al. [14] and Agopiantz et al. [13] recently reviewed IHC testing of ER, the articles reviewed in Figure 2 from Agiopiantz et al. [13] (reproduced in Figure A2), were used as the basis for the following analysis because of a more recent online search (2022 vs. 2020) and the increased detection of ER of 11.3% (95% CI: 5.9–16.7) vs. 0.03 (95% CI 0.01–0.09) by Miyagishima et al. [14]. This data series [65,83,84,85,86,87,88,89,90,91,92,93,94,95,96,97,98,99,100,101,102,103,104,105] was supplemented by the addition of three studies [106,107,108] found in Miyagishima [14] and three older studies [48,49,109] that appear in neither review (see Table A2). With these additions, the mean ER expression of the 30 studies (2331 cases) in this series is higher than that in both previous reviews at 16.43 (95%CI 8.415–24.44).

As illustrated in the depiction in Figure 3a, a major factor affecting the visual detection of the brown precipitate that marks the presence of ER is the ability or affinity of the primary antibody to recognize ER. The affinity of anti-ER antibodies improved over time from the initial H222^TM^ (Abbott ER-ICA) rat monoclonal antibody, through the two mouse monoclonal antibodies, 1D5^TM^ (DAKO, now Agilent) and 6F11^TM^ (Novocastro, now Leica), to the current standard SP1^TM^ (Roche) rabbit monoclonal antibody. Another key parameter in IHC testing is whether a specific percentage of tumor cells must be positively stained to classify a case as ER+. For breast cancers, a 10% threshold of positive cells is often used to classify a case as ER+.

In Figure 3b, the results of the 30 IHC studies are summarized, specified by antibody and whether a threshold was used in classifying a case as ER+ (details in Table A2). Based on the extracted data (Figure 3b), none of the six studies [49,65,98,99,100,101] using the anti-ER antibody H222 detected any ER+, regardless of whether a threshold of staining was used. As antibodies improved and ER assays shifted from 1D5 [85,86,88,92,102,103,107] to 6F11 [87,90,106] and then SP1 [89,91,93,94,95,96,97,105], there was a slight but non-significant improvement in ER detection over H222.

Six studies used non-standard or unspecified antibodies (see Misc column in Figure 3b), one [104] of which had a threshold for positivity and reported no ER staining. The five papers [48,83,84,108,109] without a stated threshold showed significantly increased detection of ER when compared to H222 and SP1 and included three papers using either a proprietary antibody (DFCI) [83,84] or fluorescently labeled estradiol [48]. The two papers with the highest levels of ER expression at 62% [108] and 86% [109] involved unusual populations and will be discussed separately below.

Importantly, any studies that required that the number of positively stained cells exceed a threshold ranging from 1 to 10% [65,98,99,102,103,104] did not report any ER+ cases, regardless of the antibody used. In all papers reporting ER+ cases, positives are described as “weak staining in scattered, occasional cells” which would generally be regarded as equivocal or negative for ER in a breast cancer assay. This result has contributed to the general consensus that ER and hormones in general are unimportant in ICM.

There are three complementary factors that could contribute to this low level of ER detection, and these will be addressed in the following section.

## 4. Factors Causing Low Levels of Detectable Estrogen Receptors

### 4.1. Suppression of ER Concentration by Dex

IHC studies in ICM use the same methodology as that used in breast cancer ER testing. In the 16 IHC studies [65,83,84,85,88,89,92,97,98,99,100,101,102,103,104,105] in this review that specified controls, all used tissue sections from ER+ breast cancer as positive controls. However, IHC ER testing in breast cancer is “calibrated” to detect ER levels ≥10 fmol/mg protein, a level that best predicts outcome with endocrine therapy in breast cancer [110]. Increasing the sensitivity of IHC detection results in “false positives”, whereby these low expressing breast cancers behave more like ER-negative tumors with a worse prognosis requiring chemotherapy and deriving no benefit from hormone treatments [111]. As discussed previously, LBA testing after Dex exposure suggests that approximately 12% of ICMs have ER ≥10 fmol/mg and would be expected to be ER+ by IHC. This is concordant with the overall IHC ER+ expression of 16.43 (95%CI 8.415–24.44), confirming the low level of ER expression seen in LBA studies after the widespread adoption of perioperative Dex in 1984. There are three additional studies that support Dex-induced suppression of ER levels. A total of 12/14 (86%) of ICM patients not exposed to Dex were ER+ in a 1986 IHC study [109]. In contrast, only one of four (25%) ICM patients who demonstrated pre-operative uptake of labeled estrogen (16α-[18F]-fluoro-17β-oestradiol) by Positron Emission Tomography (PET) was weakly positive according to IHC testing after surgical resection [112]. When used with breast cancers, this technique is 98% specific at detecting tissues that will be ER+ according to IHC testing [113]. Finally, an ultra-sensitive DNA-binding assay was able to detect ER protein in 14/15 (93%) of ICMs, including 10 ER-PR+ and 3 ER-PR- by LBA, suggesting that negative ER detected by LBA and IHC testing may represent an assay sensitivity problem [114].

### 4.2. Inhibition of ER Signaling by Dex

The IHC studies also reveal that ER staining is unrelated to PR [14,86,93] or pS2, another estrogen-modulated protein [115], and shows no evidence of ER signaling [86]. These findings suggest that the residual ER detected by IHC is not transcriptionally active. Of interest, residual ER staining is associated with a worse prognosis [93,108], a higher grade [92,93,94,108] and an increase in abnormal karyotypes [93]. A study of 87 WHO grade III ICMs resected between 2003 and 2008 and likely Dex-exposed reported categorically different staining percentages of ER and PR of 62% and 15%, respectively [108]. Since estrogen ligation of ER is normally followed by autoregulatory downregulation of ER [116], it is possible that the elevated levels of ER seen in high-grade ICM also represents failure of ER transcription to downregulate ER.

### 4.3. Endogenously Low Levels of ER in ICM

The most sensitive and specific method of detecting ER is the reverse-transcriptase polymerase chain reaction (RT-PCR), which detects ER mRNA [117]. Four such studies found ER mRNA in 74/85 (87%) of ICMs despite sub-threshold values with conventional LBA or IHC testing [83,114,118,119].

**Figure 4:**
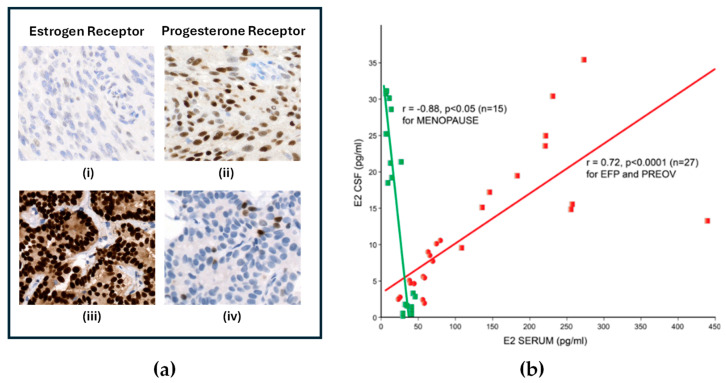
(**a**) Immunohistochemistry of ER (**i**,**iii**) and PR (**ii**,**iv**) in a woman with meningioma (**i**,**ii**) and breast cancer (**iii**,**iv**). Original objective magnification 20×. Photomicrographs provided by Dr. B. Adam (Meningioma) and Dr. G. Bigras (Breast Cancer) with patient consent. (**b**) Estradiol levels: CSF to serum correlation for fertile (n = 27, red) and menopausal women (n = 16 green). Red circles—values for Early Follicular Phase (EFP); red squares—values for Preovulatory Phase (PREOV). Green square—values for post-menopause (MENOPAUSE). Redrawn from Lutescu, I. et al. 2007, Figure 1 [123] and used with the author’s permission.

Even though the majority of ICMs may have ER mRNA, data from pre-1984 LBA studies suggest that the mean ER protein concentration even without Dex is probably less than 12 fmol/mg or 15% of the average ER+ breast cancer value. This has resulted in situations such as the one documented by Magdelenat et al. [120] and illustrated below in Figure 4a from another patient. In the 1986 report, a 59-year-old woman with a 19-year history of a frontal falx ICM with multiple recurrences presented with a new ICM in the parieto-occipital region along with synchronous breast cancer. Testing of the two new primaries found the ICM to be ER borderline positive and PR strong, while the breast cancer was strongly positive for ER with PR below the threshold. The circulating serum estradiol was characteristic of a post-menopausal women at <10 pg/mL [120].

The reason for such different concentrations of tumoral ER may lie in the ER response to the different microenvironmental estrogen concentrations. Meningiomas arise from the arachnoid lining of the skull, forming the external layer of the sub-arachnoid space for cerebrospinal fluid circulation (CSF). In post-menopausal women, while serum estradiol levels fall precipitously, crucial brain functions are maintained through the local production of brain-derived estrogen (BDE) [121] from the aromatization of free testosterone by aromatase enzyme-containing cells localized to the amygdala, hypothalamus, thalamus, and medulla [122]. Lutescu et al. [123] reported that the estradiol concentration in the CSF of post-menopausal women peaks at 30 pg/mL (0.1 nM or 10^−10^ M) (Figure 4b).

**Figure 5:**
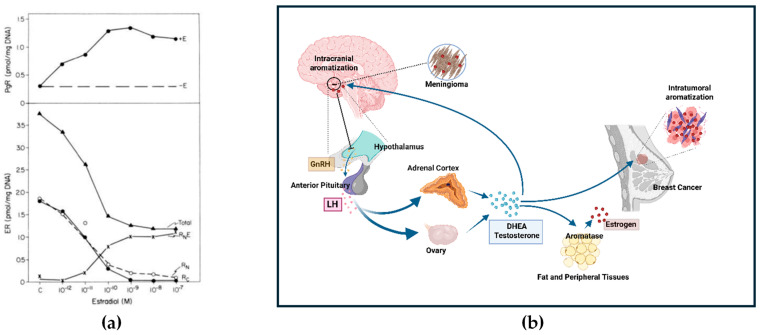
(**a**) The effect of estradiol on PR and ER distribution in MCF-7 cells. Cells were treated for 4 days with increasing estradiol concentrations (0.001 to 100 nM) added to minimal essential medium containing stripped calf serum, insulin, hydrocortisone, and prolactin. Unoccupied cytoplasmic receptors (●, Rc), unoccupied nuclear receptors (ο, Rn), occupied nuclear receptor (x, RnE), and total cell receptors (Rc + Rn + RnE). (Originally published by Horwitz K. and McGuire W, 1978, Figure 3 [124]. Used under Creative Commons CC-BY License). (**b**) Hypothetical schema for breast and meningioma malignancies. The Kisspeptin-Neurokinin B-Dynorphin (KNDy) neuronal network responds to BDE levels by modulating the release of Gonadotropin-Releasing Hormone (GnRH) from the hypothalamus which in turn stimulates the release of Luteinizing Hormone (LH) from the anterior pituitary. This shifts the adrenal cortex and ovary (in early post-menopause) towards increased C-19 steroid production of Dehydroepiandrosterone (DHEA), other androgenic pro-hormones and testosterone. Circulating testosterone is aromatized to estrogen in fat, peripheral tissues and peritumoral cells surrounding tumors in the breast and brain. Created in BioRender. Hugh, J. (2026). https://BioRender.com/2ubvxwg, accessed on 10 January 2026.

In vitro work with estrogen-deprived MCF-7 ER+ breast cancer cells found that the addition of 0.1 nM estradiol is associated with maximal induction of PR and a reduction in total ER levels due to the disappearance of unoccupied cytoplasmic and nuclear receptors with little accumulation of occupied nuclear receptors (see Figure 5a for results of 4-day estradiol stimulation). This is followed by a progressive 70% reduction in total ER compared to unstimulated cells [124]. This could explain the strong staining for PR (see Figure 4(aii)) while the endogenously low levels of ER, which would be further suppressed by Dex administration, result in low or undetectable ER by a subsequent IHC test (see Figure 4(ai). In vitro, the removal of estrogen causes PR levels to decrease to control levels after 4 to 6 days [124]. A similar delay in PR levels returning to ER-independent levels could explain the persistence of PR in ICM despite perioperative Dex and the subsequent rapid decline in PR in ex vivo cultures [115].

In contrast, the intra-tumoral estrogen concentration in ER+ breast cancers is 8-fold increased (approximately 1 nM or 10^−9^ M) over circulating serum levels [125] due to the aromatization of serum testosterone by the peritumoral tissue [126,127,128]. Horwitz and McGuire found that chronically elevated levels of estradiol of 1 nM or more would result in depletion of unoccupied cytoplasmic and nuclear receptors, leaving moderate to high levels of exclusively receptor-bound ER in the nucleus [124]. This in vitro finding is consistent with biochemical calculations of ER+ breast cancers [129]. Thus, this level of estrogen stimulation in the breast tumor microenvironment would result in nuclear ER levels which would be easily detectable by diagnostic tests (see Figure 4(aiii)).

A schematic is presented in Figure 5b. To the authors’ knowledge, this is the first report of a possible link between BDE and ICM.

## 5. Estrogen Receptor-Based Therapy: Past and Future

Corroborative evidence for functional ER in ICM includes the widespread presence of PR in low-grade meningiomas. PR is regulated by estrogen [130] via liganded ERα binding to estrogen response elements on DNA surrounding the PR gene [15] and is used clinically in breast cancer as an indicator that the ER axis is functional [131]. Although ER-PR+ breast cancers were reported by McGuire WL et al. in 1977 to constitute 9% of all ER- and 2.3% of all breast cancers [131], the frequency of ER-PR+ approaches zero in breast cancer with improved IHC assays [132]; so, ER-PR+ breast cancers are widely regarded as an anomaly.

Given this dependency of PR on ER and the assumption that ER must be functional, there have been two clinical [16,17] and three registry-based [133,134,135] studies of the partial estrogen agonist/antagonist Tamoxifen (Tam) in ICM (Table 1). The clinical studies of Markwalder [16] and Goodwin [17] showing an immediate progression rate of 30–50% and the transient nature of responses discouraged further investigations into Tam. However, the patients in these two studies consisted of inoperable or refractory meningiomas with negative or unknown ER status. These patients are similar to the 1981 pivotal trial of Tam in unselected (hormone receptor status unknown in 80%), post-menopausal women with progressive metastatic breast cancer [136] where the median time to treatment failure on Tam was 171 days (5.7 months) in 81% of patients. Yet that study was the foundation for Tam’s dominance as the standard hormone therapy in ER+ breast cancer for many years [137].

Two of the three population registry studies investigated cohorts of breast cancer patients who subsequently developed meningiomas [134,135]. Both found that breast cancer patients taking Tam significantly lowered their incidence of subsequent meningiomas compared to breast cancer patients who were not taking Tam. There was also a protective effect of Tam with a dose–response relationship in both studies (significant in [135]), whereby longer durations and/or higher cumulative doses of Tam were associated with a lower relative risk of meningioma. In contrast, the French retrospective case–control study of meningioma patients that were treated with Tam did not find a significant difference in overall survival or progression-free survival with Tam at 10 years [133]. They also found a significant progression free survival improvement in patients receiving only pre-operative Tam (log-rank *p* = 0.029) that was not further addressed. The critical limitations of this study are the lack of individual patient data regarding the grade of resection, the reason for Tam treatment (possibly indicative of a poorer prognosis or inoperability) and the short duration of Tam treatment (1.7 years) compared to the 10-year follow-up.

Although it is difficult to summarize neatly, the existing data on Tam show some albeit equivocal support for its use in ICM.

The incidence of ICM with hormone replacement therapy (HRT) is also instructive. Three studies [138,139,140] reported an increased risk of ICM with estrogen plus progesterone (E + P)-containing HRT regimens. Although two studies [138,141] found a lower but significant risk of ICM with estrogen-only (ET) HRT, this is less convincing since two other publications using the same data sources [140,142] found no evidence of a significantly increased risk with ET. Paradoxically, two studies show a beneficial effect of ET: Custer et al. [107] in a sub-analysis of their case–control report described a decreased meningioma risk with ET if given greater than 10 years after menopause. Similarly, a retrospective case–control study of patients with incidental ICM followed expectantly with and without ET found that ET treatment was associated with significantly lower growth rates and a longer clinical progression-free interval [143].

The differential effect of combined E + P compared to ET in older women is reminiscent of the finding of the Women’s Health Initiative clinical trials [144]. The 20-year follow-up of these randomized placebo-controlled trials with an average enrollment age of 63 years found that conjugated equine estrogen (CEE) plus medroxy-progesterone acetate (MPA) had an increased risk of subsequent breast cancer (HR = 1.28 (1.13–1.45)) whereas CEE alone had a decreased risk (HR = 0.78 (0.65–0.93)) of low-grade breast cancers. This suggests that low-grade breast cancers, like normal breast tissue [145,146], may not proliferate in response to estrogen. It remains to be seen if a similar relationship with estrogen exists in low-grade ICM.

## 6. Recommendations

### 6.1. Replace Dexamethasone in ICM

Given that Dex is less effective in ICM, serious consideration should be given to replacing its use with alternatives such as Bevacizumab, a humanized monoclonal antibody against VEGF that prevents binding to its receptor, or VEGFR (VEGF receptor) inhibitors such as sunitinib or vatalanib. Anecdotally and in clinical trials, Bevacizumab has decreased PTBE [147], while all three agents targeting VEGF have shown anti-tumor effects in refractory grade II/III meningiomas [148,149,150].

### 6.2. ER Testing in ICM

Reports of ER testing with IHC should indicate the antibody, any use of a threshold, Dex exposure and whether the IHC protocol is the same as that used for breast cancer. Tissues from patients with no Dex exposure should be tested for ER to confirm the presence of ER. Dedicated ER assays with increased sensitivity without sacrificing specificity should be developed for ICM.

### 6.3. Analysis of the WHI for Meningiomas

The WHI trial of post-menopausal estrogen-only replacement therapy should be investigated [144] for differences in the incidence of ICM in the placebo vs. the ET arms. The older age at recruitment avoids peri-menopausal hormonal fluctuations and may yield sufficient numbers for a meaningful analysis given the incidence of meningiomas in a similarly aged population reported in the Iowa Women’s Health Study [151]. Such an analysis could (i) reveal if estrogen could aid in the prevention of meningiomas in older post-menopausal women and by extension (ii) determine if the existing tentative evidence for Tamoxifen highlights its anti-estrogen or weak estrogenic effects, as this would direct the search for future hormonal therapies for meningioma, and finally (iii) identify what kind of breast cancer is most associated with meningioma.

### 6.4. Clinical Research

There should be new clinical trials investigating hormonal therapy in ER+ ICM patients. If ER cannot be detected reliably, then proxies such as PR+, low-grade or meningothelial histology could be enrolled. One possibility for a prospective randomized controlled trial would enroll post-menopausal women with previously resected low-grade PR+ skull-based meningiomas which have recurred. The effect of hormones vs. placebo could be monitored during the course of their regular MRI follow-ups, similar to the approach used by Dresser et al. [143]. If recruitment is low, patients could serve as their own controls in a cross-over design.

### 6.5. Prevention

There should be a registry of patients with both meningioma and breast cancer. In addition to revealing which patients might be at risk for a subsequent tumor, such a registry may find unexpected relationships between the grades of meningioma and breast cancer in a single patient that could shed light on shared hormonal etiological factors.

## 7. Conclusions

Despite the paucity of detectable ER, the incidence and growth of ICM are strongly influenced by female sex hormones. A review of ER testing suggests that since 1984, the routine use of Dex has lowered ER levels and interfered with ER signaling. This interference, combined with an initially lower level of ER, has resulted in a catastrophic underestimation of the role of hormones in this tumor. Even though the detection of ER has lagged, there is some evidence that estrogen-targeted therapies should be considered. It is time to restrict or replace Dex given its immunosuppressive effect, its reduced efficacy for PTBE in meningiomas, and the high probability that it is interfering with ER detection. Specific recommendations were made for improved ER testing as well as research into estrogen-targeted therapies for conservative management and prevention of ICM and/or associated breast cancer.

## Figures and Tables

**Table 1:** Clinical trials of Tamoxifen in ICM.

Author, Year [Ref]	Study Design	No. of Pts/Time *	Outcome	Comments
Markwalder, T., 1985 [16]	Pilot study of inoperable pts	6 pts/12 m	1—partial response 2—transient response 1—stable 2—progressed	Diagnosis unconfirmed (2) 1 case ER-PR+, 5 unknown. no grade given
Goodwin, J., 1993 [17]	Phase II of inoperable/refractory pts	18 pts/31 m	1—partial 2—transient 6—stable 9—progressed	No histologic type or grade ER neg (1) or unknown (17) No PR status.
Champeaux-Depond, C., 2021 [133]	Retrospective case-control (SNDS), matched for age, site, grade	251/1.5 y	No significant difference in OS or PFS with Tam at 10 yrs.	105 cases with 1.7 y pre-op TAM only had better PFS (*p* = 0.029) No data on ER, PR, quality of resection or treatment rationale
Ji, J., 2016 [134]	Historical population cohort of breast cancer pts developing meningioma relative to general population	223/	SIR prior to 1987 (“no Tam”) = 1.54 (95% CI 1.3–1.81) vs. SIR after 1987 (“Tam”) = 1.06 (95% CI 0.84–1.32)	No patient level data or control for grade, ER, PR status or Tam exposure. Notes that this would bias results to the null.
Sun, L.M., 2019 [135]	Population based cohort of breast ca pts with meningioma 2000–2008	42 Tam vs. 35 no Tam	HR = 0.64 (95%CI 0.40–1.02) HR = 0.42 (95%CI 0.19–0.91) If Tam for over 4 y (8 pts)	Major assumption that meningioma risk is constant regardless of ER status of breast cancer.

* No. of Pts/Time—number of meningioma patients treated with TAM/median time of treatment (m—months or y—years). SNDS—Système National des Données de Santé, SIR—standardized incidence ratios, and HR—hazard ratio.

## Data Availability

No new data were created or analyzed in this study. Data sharing is not applicable to this article.

## Appendix A

**Table A1:** Comparison of % of cases above threshold and mean ± SEM assay value in cases with and without Dex exposure.

First Author, Year [Ref]	Dex Exposed % Cases (n) Above Threshold	No Dex % Cases (n) Above Threshold	Dex Exposed Mean ± SEM All Cases	No Dex Mean ± SEM All Cases
Expressed in fmol/mgm of protein				
Schwartz, M. 1984 [46]	26 (6/23)	67 (2/3)	35.5 ± 21.2	76 ± 66
Yu, Z. 1982 [47]	10 (1/10)	50 (3/6)	4.9 ± 1.1	10.6 ± 2.8
Expressed in fmol/gm of Tumor				
Magdelenat, H. 1982 * [76]	75 (12/16)	91 (10/11)	166 ± 29	339 ± 57
Poisson, M. 1983 [78]	28 (5/18)	50 (3/6)	121.7 ± 61.3	201.7 ± 117

* No Dex cases include 11 women where only 1 received Dex and Dex exposed cases include 16 women, 8 of whom received Dex.

**Table A2:** Expression of estrogen receptors by immunohistochemistry. Summary data presented in Figure 3b.

Author, Year	Anti-ER Antibody	Threshold Used	% ER Positive (n/no.of Cases)	Ref
Bozzetti, C. 1995	H222	>10%	0 (0/44)	[65]
Brandis, A. 1993	H222	>10%	0 (0/61)	[98]
Bouillot, P. 1994	H222	>5%	0 (0/52)	[99]
Halper, J. 1989	H222	None	0 (0/47)	[49]
Khalid, H. 1994	H222	None	0 (0/34)	[100]
Perrot-Aplanat, M. 1992	H222	None	0 (0/36)	[101]
Nagashima, G. 1995	1D5	Scored	0 (0/39)	[102]
Hirota, Y. 2009	1D5	>10%	0 (0/82)	[103]
Konstantinidou, A. 2003	1D5	None	35 (17/48)	[85]
Claus, E. 2008	DAKO	None	33 (10/31)	[86]
Leaes, C. 2010	1D5	None	25 (32/126)	[88]
Hsu, D. 1997	1D5	None	8 (6/70)	[92]
Custer, B. 2006	1D5	None	1 (2/143)	[107]
Korhonen, K. 2006	6F11	None	40 (159/510)	[106]
Guevera, P. 2010	6F11	None	28 (12/42)	[87]
Takei, H. 2008	6F11	None	10 (6/57)	[90]
Iplikcioglu, A. 2014	SP1	Scored	0 (0/48)	[105]
Giraldi, L. 2021	SP1	None	20 (19/97))	[89]
Shahin, M. 2019	SP1	None	9 (1/11)	[91]
Pravdenkova, S. 2006	SP1	None	7.5 (18/239)	[93]
Kuroi, Y. 2018	SP1	None	7.5 (12/161)	[94]
Portet, S. 2020	SP1	None	5 (3/60)	[95]
Abdelzaher, E. 2011	SP1	None	3 (2/60)	[96]
Telugu, R. 2018	SP1	None	2 (2/100)	[97]
Heβ, K. 2017	M7047	Scored	0 (0/89)	[104]
Carroll, R. 2000	DFCI	None	43 (9/21)	[84]
Carroll, R. 1999	DFCI	None	38 (10/26)	[83]
Hinton, D. 1983	Estradiol	None	27 (3/11)	[48]
Hua, L. 2018	Unk	None	62 (54/87)	[108]
Maiuri, F. 1986	JS 34/32	None	86 (12/14)	[109]

Data were extracted from the tables in each reference. Where thresholds have not been explicitly identified, results characterizing positive cases as showing only scattered and occasional cells staining, are taken as “no threshold”.
